# A Review on Glaucoma Drainage Devices and its Complications

**DOI:** 10.7759/cureus.29072

**Published:** 2022-09-12

**Authors:** Sajal Gupta, Sandhya Jeria

**Affiliations:** 1 Ophthalmology, Jawaharlal Nehru Medical College, Datta Meghe Institute Of Medical Sciences, Wardha, IND

**Keywords:** ahmed, krupin, baerveldt, molteno, glaucoma, i.o.p, g.d.d

## Abstract

Glaucoma is one of the leading causes of blindness in the world. It is an ocular disorder that may have multiple etiologies and which can present as optic neuropathy and increased intraocular pressure (IOP), but in some cases like normotensive glaucoma, the IOP may remain normal. Its gradually progressive nature makes it important for early diagnosis; although the loss of vision is slow, lost vision can't be restored. Glaucoma drainage implant surgeries are an increasingly popular option in recent days in complicated cases of glaucoma where the previous trabeculectomy had failed and medical management was not responsive. Glaucoma drainage devices (GDD) are of various designs; they are implanted according to the patient condition and surgeons' preference. There are complications after the implantation of a GDD like hypotony, endophthalmitis, migration of the plate, extrusion, erosion of the mucous membrane, etc. In the market, there are various drainage devices present, but some of them are frequently used and popular.

In this article, we will discuss some most commonly used GDDs and their complications. Among these, four are the most popular: Molteno, Baerveldt, Krupin, and Ahmed. The failure rate of the GDD is low. In many studies, it has been noted that only half of the GDD remains functional after five years. Therefore, further studies are still being conducted to refine the biomaterials, techniques, and shape of the GDD. The technique of surgery is also very much crucial in the success of GDD implantation. The glaucoma type is an essential factor in deciding the treatment, and the outcome of the surgery also depends on it.

## Introduction and background

Glaucoma is a progressive and chronic neuropathy of the optic nerve which many ocular conditions may cause; these may lead to loss of vision. Among these, the most common risk factor that can lead to vision loss is raised intraocular pressure (IOP) [1]. The factors associated with glaucoma are old age, any history of glaucoma in the family, and some races [2]. In most cases, the condition is asymptomatic, so it is generally undetected in the early phase of the disease. So, the physician must screen all high-risk cases to avoid irreversible blindness. There are many conditions that ultimately lead to optic neuropathy which is known as glaucomatous optic neuropathy (GON). Various reports are suggestive that the primary site of injury is the optic nerve head [3].

There are so many reasons for the increase in IOP, among these most common reasons are: (1) increased rate of formation of IOP, (2) or difficulty in its drainage, and (3) rise in the pressure of the episcleral vein [4]. In most cases, the pressure rise is due to the increasing resistance in the drainage of the aqueous humour through the angle of the anterior chamber and/or due to the circulation of aqueous humour at the pupil. All these points should be kept in mind while choosing the implants for glaucoma drainage because the knowledge of the specific cause is very important while giving any treatment to the patient, particularly while placing implants [5]. The two main pathologies that occur in glaucoma are: (1) mechanical changes due to the increased IOP and (2) decreased perfusion of the optic nerve head [6]. Any previous damage cannot be reversed, but by placing the drainage devices further damage can be prevented.

Glaucoma is traditionally divided into primary and secondary glaucoma. Primary adult glaucoma is further divided into open-angle and angle-closure glaucoma. Secondary glaucoma is due to any disease of the eye. Primary open-angle glaucoma can be treated by medical management like prostaglandin and beta-blockers but if this medical treatment fails to manage the cause then laser trabeculoplasty is a good option. Primary angle-closure glaucoma can be managed by Nd:YAG iridotomy in both of the eyes and after that the patient has to get the IOP checked at regular intervals for the rest of their life. Secondary open-angle glaucoma can be managed by medical therapy but if it fails to control the symptoms, then surgery is the option for its treatment. The treatment for secondary angle-closure glaucoma is by controlling the inflammation and IOP through medical therapy, and congenital glaucoma can be managed by goniotomy and trabeculectomy [7].

Treatment of glaucoma can be done either by medications (topical or systemic IOP reducing agent), laser, or surgery. In the last few years, many medicines for the treatment of glaucoma have come on the market, and instead of invasive incisional surgery, the use of trabeculoplasty (laser) is increased [8]. The GDD are the devices that are used to drain the aqueous from the anterior chamber to the reservoir, which is formed externally weeks after the surgery by which the flow is maintained. These devices have shown success in cases in which there is scarring in the cornea due to previous ocular surgeries or in the cases of failed trabeculectomy [9]. Glaucoma can be controlled by medications, laser surgeries, and trabeculectomy. Nowadays, implants for drainage of glaucoma are also good options. As in many studies, it has been noted that these devices successfully control the IOP in those cases in which previous trabeculectomy failed. Various devices have been developed in the market according to the need for surgeries. Preoperative IOP, the status of the optic nerve, and the surgeon's preference are all important factors in choosing the implant [10]. The first glaucoma drainage was done by Zorab using silk thread to drain glaucoma [11]. 

**Figure 1:**
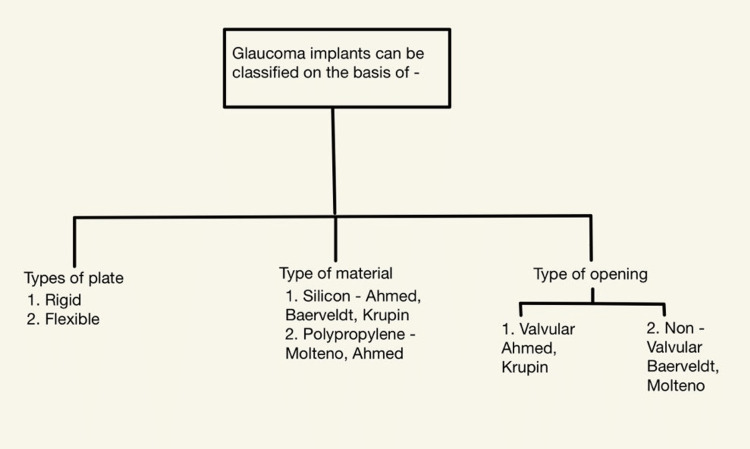
Classification of glaucoma implant devices

The GDD is classified according to the (1) type of material: the glaucoma drainage devices are made up of materials such as silicon and polypropylene; the silicone implants are Baerveldt, Krupin, and Ahmed and the polypropylene implants are Ahmed and Molteno; (2) type of opening: The glaucoma drainage devices are either valvular or non-valvular. The valvular implants are: Krupin, Ahmed [12]. The non-valvular implants are: Molteno, Baerveldt (Figure 1) shows the classification of GDD.

## Review

Different implants are developed from time to time after observing the complications of the previously present device. As different implants are developed, modifications are made according to the complications of the previous one. Table 1 shows the year of development of different implants and their postoperative complications.

**Table 1:** Year of development of different Implants and their postoperative complications

Name of glaucoma drainage devices.	Year of development	Types of opening	Postoperative complications.
Molteno	1979	Non-valvular	More chances of hypotony.
Baerveldt	1990	Non-valvular	More chances of hypotony. Greatest amount of chances of diplopia are found with this implant.
Krupin	1990	Valvular	Comparatively fewer chances of hypotony
Ahmed	1993	Valvular	Comparatively fewer chances of hypotony. Choroidal effusion.

Molteno implant

The first device which was used to drain glaucoma was the Molteno implant. This GDD was first given in 1969. Basically gives the basic idea on which all the GDDs of the present date are based [13]. As the years passed, several modifications have been made to its original design, and advancement in the technique of surgery has led it to tremendous success and lower complication rates. The Molteno implant is a non-valvular device that consists of a tube that is made up of silicon and attached to a tube placed 9-10mm posterior to the limbus within subconjunctival space. The plate of the Molteno implant is sutured to the sclera and then covered by tenon tissue and conjunctiva, a bleb is formed over it, which is fibrovascular and permeable; its surface area contributes to the amount of aqueous drainage and final IOP [13]. When described in detail, this implant consists of a thin tube with an outer diameter of 0.6mm and an inner diameter of 0.3.mm, which opens up onto the acrylic plate, which is 13 mm in diameter, thin and circular. The plate has an edge rim of 0.7mm thick that is also perforated for attachment of it with sclera by sutures which prevents it from dislocation [14].

Original device modification of Molteno device

In the recent era, the Molteno implant device has been modified to a double plate model, which helps in increasing the potential space for aqueous absorption. This double plate model consists of two plates of 13mm diameter and a 10mm diameter tube connecting them. The double plate model demands more skills and technique than the single plate model. This model doubles the surface area for the absorption of aqueous. It is also found that the two-plate model provides better drainage of the aqueous than the plate as well as the four-plate model [14].

Complications of Molteno device implantation surgery

The Molteno implant device is widely used in refractory glaucoma. However, it has its own complications; the modifications in surgical techniques like ligation of tube temporarily, for insertion of the tube the needle track is used, and a scleral patch of the donor reduce some of them but not eliminate them. In some patients with neovascular glaucoma, postoperative hyphema has been reported. Still, this condition is generally resolved within days of its occurrence and doesn't affect the result of the surgery. In some cases, the hypotony and shallow anterior chamber has been reported due to the aqueous leakage around the silicon tube. Some other severe complications have also been reported, like vitreous haemorrhage and retinal detachment, but in very few cases [15]. The Molteno implant help in draining the aqueous humour from the anterior chamber to the reservoir posteriorly, so it becomes a good option in the management of refractory glaucoma. The problems which were associated with the Molteno implant are reduced by the change in surgical technique and design modification of the implant.

Baerveldt implant

In 1990 this implant was introduced for the management of recalcitrant glaucoma surgically. This implant has only one plate without any valve, which restricts the flow; because of this reason, ligation of the tube in surgical procedures is essential for making sufficient space for the absorption of fluid [16]. Presently the available Baerveldt glaucoma drainage device (BGDD) is superior to the earlier implants in two ways: first, the implantation process is relatively easy, and second, the surface area is large (the reason why surface area makes it better is that the diffusion of aqueous humour is by the wall of bleb enfolding the implant, and also IOP lowering is proportional to the area of bleb). The increased surface area of the BGDD in comparison to a single small plate Molteno device describes its lower, longer control on pressure [17].

BGDD is commonly placed between the two rectus muscles, generally lateral and superior or inferior and medial; this requires dissection of conjunctiva on one quadrant only [17]. The non-valvular BGDD tube may help to maintain low resistance to the flow, thereby maintaining the low IOP for a long time [17]. This implant is non-valvular and consists of impregnated barium and rounded silicon having a surface area of 250-350 mm2 [18]. This implant has many features similar to the Molteno implant, but the only difference is it is made of soft silicon [19]. BGDD was found to be effective in decreasing intraocular pressure in patients suffering from intractable glaucoma [20]. This implant is also effective and safe in treating glaucoma with uveitis [21]. Implantation of BGDD inferonasal was effective and secure and can be helpful in some instances [22]. Initially, this implant showed an excellent success rate, but it is seen that its rate of survival decreases with time. In most cases, this requires numerous surgeries so that IOP can be maintained and vision can be preserved [23]. The BGDD is suitable for treating high intraocular pressure due to refractory glaucoma. In the cases where the management of glaucoma is difficult, like in the cases of neovascularization of the iris, scarring in the conjunctiva, etc. The surgery should be done by careful planning, and attention to the surgical technique can reduce postsurgical complications.

Complications of BGDD

According to the studies, the incidence of corneal edema is highest in postoperative cases, and tube complications are the next most common complication, including occlusion of the tube, erosion of the tube, and malposition of the tube. Motility disorder (2-17%), diplopia, and hyphema (2-19%) are the other postoperative complications that can happen [24]. Severe complications after surgery like blebitis (1-2%) and retinal detachment (1-2%) are significantly less. In about 6% of the cases, no perception of light was found postoperatively [25].

Aurolab aqueous drainage implant (AADI)

Aurolab aqueous drainage implant (AADI) was developed by Aurolab in Madurai, India. It has been available commercially in the Indian market since 2013. The surface area of AADI is 350mm2; it has lateral wings, which are designed in such a way that it gets its position under the rectus muscle. The fixation holes are present that help the end plate anchor 10mm posterior to the limbus [26]. It is a non-valvular GDD and its structure is based on BGDD. The best benefit of this implant is it is very cost-effective compared to the Ahmed implant and so very profitable in developing countries where the incidence of pediatric glaucoma is high [27].

This implant provides a large surface area, which helps to attain low IOP as compared to the devices with valves. The chances of encapsulation of the bleb are significantly less because it is not directly exposed to the mediators of inflammation from the anterior chamber; it is only in the case if the tube is opened up, which is most likely after four to six weeks [28]. So, there is no such hypertensive phase as that in valvular devices, but in the initial weeks, there may be the chance of episodes of increased IOP until the ligatures give way. At the initial time, the patient should be on medications so as to control the IOP [29].

Krupin implant

The concept of the Krupin implant was first developed in 1974. This implant is made up of a Silastic tube that is open, which has an outside diameter of 0.58mm and an inside diameter of 0.38mm. The length of this tube is 20mm, and the length can be shortened at the time of surgery during its placement in the anterior chamber. The vertical and horizontal slits are present at the distal end of the Silastic tube, which is responsible for the unidirectional, pressure-sensitive valve [30]. The criteria for the calibration of manometry are the opening and closing pressure. The former is 10-12 mmhg, and the latter is 8-10 mmHg. The portion of the device, which is in the episcleral part, is an oval Silastic disc, 13*18 mm, with a 1.75 mm side wall. The shape of the disc is such that it fixes to the globe curvature [31].

Ahmed glaucoma valve

It was proposed by Mateen Ahmed, and the US Food and Drug Administration approved it in 1993 [32]. It is made of three parts: (1) plate, which can be made up of polypropylene, silicone, or polyethene (porous); (2) drainage tube made up of silicone; (3) valve, which is also made of silicone [32]. The Ahmed glaucoma valve (AGV) comes in two forms: (1) adult (S2), which has a surface area of 180 mm2, and (2) paediatric (S3) has a surface area of 96 mm2.

This implant is present in two versions one is rigid, and the other is flexible. The rigid is made up of polymethylmethacrylate, and the flexible one is made up of silicone rubber both are present in either one or two plate models. These also contain valves that restrict the flow, which helps to prevent hypotony postoperatively. It has been reported that AGV, made up of silicon, is more effective in controlling the IOP But also associated with more risk of complications compared to the polymethylmethacrylate one [16]. Recently a new model of AGV. has been introduced: M4, which is a modification of AGV S2, which contains an identical valve mechanism, but the case is made up of porous high-density polyethene. The total area of the plate is 160 mm2, from which the surface area of pores is excluded. The pores ease the vascular and fibrotic ingrowth and resist infection [32]. AGV is a good option for secondary glaucoma. Although it is user-friendly, some surgical techniques should be learned by the surgeon before the surgery. AGV implant has benefits over non-valvular devices in terms of the easy postoperative management of glaucoma. But still, hypotony in the early postsurgical phase is a complication [33].

Complications of AGV

The AGV is designed with the target of reducing the hypotony post-surgery allowing the drainage of aqueous humour when the intraocular pressure is in the range of 8-12mmHg.There are studies that tell that these mechanisms are good in decreasing the hypotony post-surgically but are not able to eradicate it completely compared to the non-valvular implants [34]. The reason why persistent hypotony occurs after an AGV implant is not known, but some precautions should be taken during the surgery that not over-priming the tube and not excessively manipulating the valve housing [35]. The most commonly known complication of all glaucoma drainage implants and for AGV also is exposure to the tube; this is because of erosion of the conjunctiva as well as the patch that covers it. It is seen that it happens only in 2-7% of the cases in the late postsurgical period [24]. Strabismus and diplopia are also the most common postsurgical complication of generally all types of glaucoma implant devices [32].

## Conclusions

In cases where the trabeculectomy failed, glaucoma drainage devices are a good option. Since nowadays various models of different shapes and sizes are available in the market, so few things are there which should be kept in mind while deciding on the implant, for instance, the choice of surgeon, IOP before surgery and status of optic nerve before surgery. The preference for implant also depends on the area of bleb present in the implant as the area of the bleb is directly proportional to the diffusion of aqueous humour from the wall of the bleb. Some of the surgeries in the past have shown various complications, according to those new modifications have been made which are described in the article. The invention of drainage devices is a great achievement in the field of medical science, but it has its complications as well, and its the doctor's duty to manage them. The main aim of the article is to make aware clinicians and laymen alike of the advantages and disadvantages of the different types of implants.
